# Reagentless and sensitive determination of carcinoembryonic antigen based on a stable Prussian blue modified electrode†

**DOI:** 10.1039/d0ra06751b

**Published:** 2020-10-16

**Authors:** Jing Lin, Kunyin Li, Meifang Wang, Xiaohong Chen, Jiyang Liu, Hongliang Tang

**Affiliations:** Guangzhou University of Chinese Medicine Guangzhou Guangdong 510006 China; The First Affiliated Hospital of Guangxi University of Chinese Medicine Nanning 530023 China tanghongliang@gxtcmu.edu.cn; Department of Chemistry, Zhejiang Sci-Tech University 928 Second Avenue, Xiasha Higher Education Zone Hangzhou 310018 PR China liujy@zstu.edu.cn

## Abstract

Reagentless and sensitive detection of tumor biomarkers using label-free electrochemical immunosensors is highly desirable for early and effective cancer diagnosis. Herein, we present a label-free electrochemical immunoassay platform based on surface-confined Prussian blue (PB) redox probes for sensitive and reagentless determination of carcinoembryonic antigen (CEA). To facilitate the electron transfer of probes and improve sensitivity, Au nanoparticles and PB (Au–PB) are electrochemically co-deposited on a carbon nanotube (CNT) modified glassy carbon electrode (GCE). A polydopamine (pDA) layer is coated on the Au–PB nanocomposite layer *in situ* as a bifunctional linker. In addition to improving the stability of PB, pDA also provides reducibility for the preparation of gold nanoparticles, which offers an interface for anti-CEA antibody immobilization. The fabricated immunosensor has good stability and is able to reagentlessly detect CEA over a wide range (0.005–50 ng mL^−1^) with high reproducibility. Furthermore, the immunosensor was used for determination of CEA in human serum samples.

## Abstract 2

A label-free electrochemical sensor is easily fabricated based on stable and surface-confined Prussian blue for reagentless and sensitive detection of carcinoembryonic antigen.

## Graphical abstract

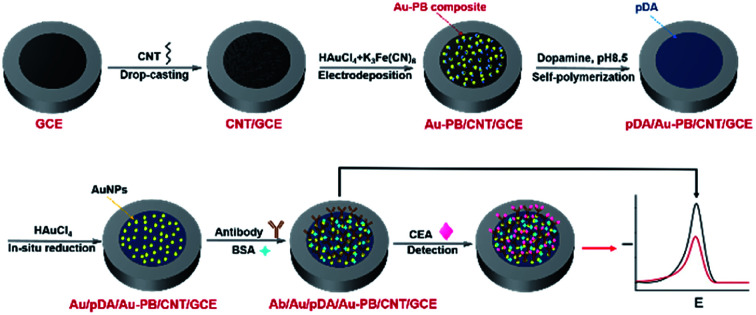

## 1. Introduction

Cancer is nowadays the greatest cause of death worldwide. Early diagnosis of cancer is critical to reduce mortality.^1–3^ As substances overexpressed in the presence of cancer in the body, cancer biomarkers are potentially valuable for early cancer diagnosis, accurate pre-treatment staging and predicting the recurrence of cancer.^4,5^ For instance, carcinoembryonic antigen (CEA) is a glycoprotein with a molecular weight of about 180 kDa. Elevated levels of CEA in biological samples (*e.g.* serum) are related to colorectal, pancreatic, liver, lung, breast, and ovarian cancer.^6,7^ Therefore, the identification of the concentration of cancer biomarkers in serum is one of the most promising strategies for early and effective cancer diagnosis. Practical approaches for simple and accurate determination of cancer biomarkers are highly desirable.

Owing to high affinity between antigens and antibodies, immunosensors have become the most important method for the detection of tumour biomarkers.^5^ Many strategies have been exploited for the fabrication of immunosensors in the past few decades, including radioimmunoassay, enzyme-linked immunosorbent assays, fluorimmunoassay, electrochemiluminescence immunoassay, gold immunochromatography assay, *etc.*^8^ Amongst, electrochemical immunosensors are particularly attractive due to their low sample consumption, simplicity, sensitivity, possibility of simultaneous multi-target analysis and potential for miniaturization or integration.^9,10^ Basically, electrochemical immunosensor can be classified into two main groups.^8^ The former usually involves covalent labelling of an affinity probe (antibody or antigen) with a redox-active reporter (label). Thus, detection is realized using sandwich immunoassay model or direct competitive immunoassays between labelled and unlabelled targets. However, the labelling process is usually time-consuming and involves complex or expensive procedures. Additionally, the labelling of an affinity probe might affect its bio-affinity towards targets. The latter form is label-free model, which can directly measure the change of electrochemical response caused by binding between antibodies and antigens. Owing to the simple operation, label-free electrochemical immunosensor has now attracted more interests.^11,12^

Signal indicator is crucial in the design and fabrication of sensitive and label-free electrochemical immunosensor. Due to the possible diffusion limit, the use of solution-phase redox probes as indicators might compromise the detection efficiency. At the same time, the free probes might bring possible side-effects including contamination to the target system or cytotoxicity to biological samples. Thus, the immobilization of redox indicators on electrode surface is efficient and has great potential in terms of the simple, sensitive and reagentless detection.^13–15^ Among the numerous redox substances, Prussian blue (PB), a coordination polymer, is one of the most representative redox probes owning to its good redox reactivity, and potential for *in situ* modification of the electrode surface.^16,17^ However, PB-based electrochemical biosensors suffer from low electron conductivity and poor stability.^18^ Fabrication of reagentless immunosensor with stable and sensitive PB signals still remains challenging.

**Fig. 1:**
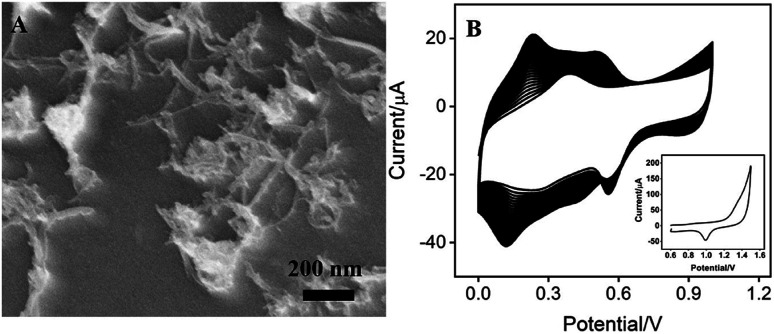
(A) SEM image of CNT/GCE. (B) CVs of CNT/GCE in KNO_3_ solution containing HAuCl_4_ and K_3_[Fe(CN)_6_] using consecutive 30 scan cycles (scan rate 50 mV s^−1^). Inset: CV of Au–PB/CNT/GCE in H_2_SO_4_ at a scan rate of 100 mV s^−1^.

In this work, we demonstrate a reagentless electrochemical immunoassay platform to detect CEA using surface-confined PB as indicator, which is easily fabricated, sensitive, and stable (Fig. 1). In this platform, multilayer structures are employed to fabricate the sensing interface with promoted electron transfer, improved stability of PB, and convenient immobilization of recognition antibody. Multiwalled carbon nanotubes (CNT) modified glassy carbon electrode (GCE) serves as the supporting electroactive architecture. PB and Au nanoparticles (Au–PB) nanocomposite prepared by electrochemically co-deposition is firstly coated on CNT/GCE, while PB is used as the redox probe and Au nanoparticles (AuNPs) facilitate electron conductivity. Subsequently, the stability of Au–PB is further improved by *in situ* polymerized polydopamine (pDA) layer, which also acts as reducing agent for the following deposition of the second layer of Au nanoparticles. Then, recognitive antibody is attached onto Au nanoparticles, which offer the recognitive interface. The principle of the reagentless detection is that the reduced signal of PB is proportional to the captured antigen on the recognitive interface. As the proof-of-concept demonstration, this immunosensor is employed to reagentlessly detect CEA. Owing to the good conductivity of CNT and Au nanoparticles, protective effect of pDA layer, and non-covalent immobilization of recognitive antibody, the as-prepared sensor demonstrates outstanding performance in terms of easy fabrication, high sensitivity, and good stability.

## 2. Experimental

### 2.1. Fabrication of label-free immunosensor

Freshly cleaned GCE was employed as the supporting electrode. Carboxylated multiwall CNT (95%, Shenzhen Nanotech. Port. Co., Ltd., China) was prepared by ultrasonic treatment in concentrated HNO_3_ and H_2_SO_4_ (v/v 1 : 3) for 6 h followed by extensive washing and filtrating. Then, 10 μL of carboxylated CNT (0.2 mg mL^−1^) was drop-coated on GCE and dried at room temperature. For the electrochemical co-deposition of PB and Au nanoparticles, CNT/GCE was immersed in a mixed solution (pH 3.2) containing K_3_[Fe(CN)_6_] (1 mM), KNO_3_ (0.1 mM) and HAuCl_4_ (1 mM). The electrodeposition was performed at 0–1 V for 30 consecutive scan cycles. Subsequently, Au–PB/CNT/GCE was immersed in an alkaline dopamine solution (0.4 mg mL^−1^ dopamine in 0.1 M phosphate buffer solution-PBS, pH 8.5) for 30 min to form PDA layer (pDA/Au–PB/CNT/GCE). Then, the second layer of Au nanoparticle was deposited on pDA/Au–PB/CNT/GCE using the reduction ability of pDA. Briefly, pDA/Au–PB/CNT/GCE was immersed in HAuCl_4_ solution (2 mM) for 2 h at 4 °C with slight stirring. The obtained Au/pDA/Au–PB/CNT/GCE was employed for the binding of recognitive anti-CEA antibody (Ab). Briefly, the electrode was incubated in anti-CEA antibody solution (20 μg mL^−1^) at 37 °C for 30 min followed with thorough rinsing with PBS (0.1 M, pH 7.4). The obtained Ab/Au/pDA/Au–PB/CNT/GCE electrode was then incubated in BSA solution (2 mg mL^−1^) at 37 °C for 30 min to block the non-specific binding sites. After thorough rising, the immunosensor was obtained and stored at 4 °C when not used. For the detection of CEA, the immunosensor was incubated with different concentration of CEA for 30 min at room temperature and the electrochemical signal of surface-confined PB was then measured.

### 2.2. Characterization

Scanning electron microscopy (SEM) and energy dispersive spectrum (EDS) was conducted on a field-emission scanning electron microscopy (S-4800, Hitachi, Japan). Cyclic voltammetry (CV) and differential pulse voltammetry (DPV) measurements were performed on a CHI 660D electrochemical station (Shanghai CH Instruments, China). Electrochemical impedance spectroscopy (EIS) was carried out on an Autolab PGSTAT302N electrochemical workstation (Metrohm, Switzerland). A conventional three-electrode system was employed in electrochemical experiments. Bare or modified GCE acts as the working electrode, Ag/AgCl electrode saturated with KCl is the reference electrode and platinum disk (1 cm × 1 cm) is the auxiliary electrode. KNO_3_ solution (0.1 M, pH 7.0) is used as the electrolyte solution because K^+^ is involved in the electrochemical process of PB based electrode through conversion between PB (K[Fe^3+^Fe^2+^(CN)_6_]) and Prussian white (PW, K_2_[Fe^2+^Fe^2+^(CN)_6_]).

## 3. Results and discussion

### 3.1. Co-deposition of PB and AuNPs on CNT modified GCE

**Scheme 1:**
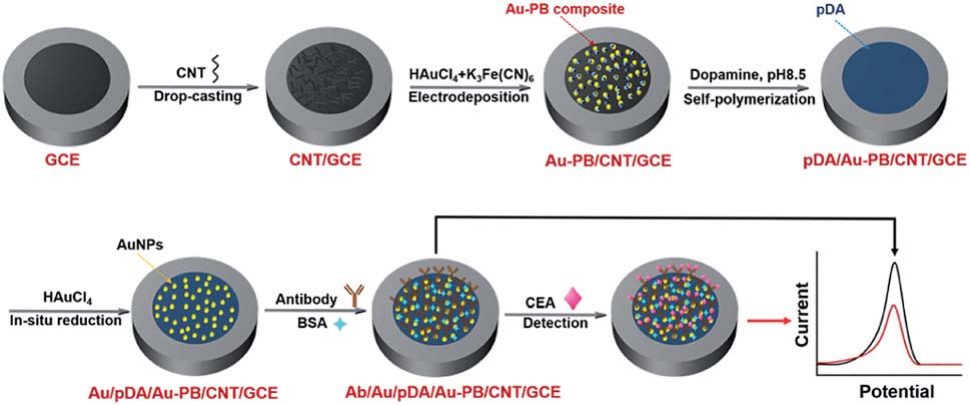
Schematic illustration for the fabrication of label-free electrochemical immunosensor and reagentless detection of CEA.

The surface confinement of electroactive mediator is crucial for the fabrication of label-free electrochemical immunosensor. As shown in Scheme 1, PB (K[Fe^3+^Fe^2+^(CN)_6_]), one of the most important and interesting mixed-valent iron compounds, is chosen because of unique merits of easy preparation, low cost, and inherent redox characteristics. Glassy carbon electrode (GCE), the most commonly supporting electrode, is used as the supporting electrode for the fabrication of label-free electrochemical immunosensor for reagentless detection of CEA. In order to further improve the electrode area and conductivity, carbon nanotubes (CNT) were drop-coated on the surface of the GCE. It is well known that CNT has nanoscaled sp^2^ carbon structure and been widely employed in the construction of electrochemical biosensors owning to high conductivity.

As demonstrated in Fig. 1A, CNT covers the surface of GCE, which is expected to increases the amount and electron transfer of PB. PB could be efficiently prepared using electrodeposition, which offers potential for *in situ* functionalization of electrode. To further facilitate the electrical conductivity, AuNPs are electrochemically co-electrodeposited with PB. The deposition process was investigated using cyclic voltammetry (CV). Fig. 1B demonstrated the typical cyclic voltammograms (CVs) for the deposition of PB. The redox couple between 0.2–0.3 V is related to the transition between PB to Prussian white (PW, K_2_[Fe^2+^Fe^2+^(CN)_6_]), indicating the formation of PB in the electrochemical deposition.^19,20^ At the same time, the obtained electrode shows the characteristic redox signal of Au in sulfuric acid medium (inset in Fig. 1B), suggesting that AuNPs are successfully co-deposited with PB on the electrode. Thus, the obtained electrode is defined as Au–PB/CNT/GCE.

**Fig. 2:**
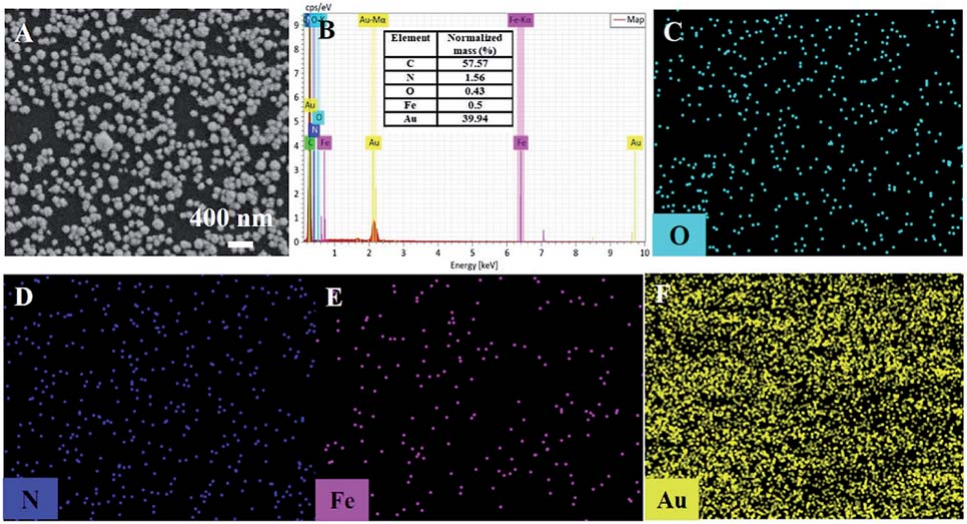
SEM image (A), EDS (B) and elemental mapping images (C–F) of Au–PB/CNT/GCE.

The morphology of the modified electrode was investigated by scanning electron microscopy (SEM). As shown in Fig. 2A, a large number of evenly distributed nanoparticles appear on electrode surface. C atoms (57.57%) from CNT or GCE, N (1.56%) and Fe (0.5%) atoms form PB (K[Fe^3+^Fe^2+^(CN)_6_]), and Au atoms from Au nanoparticles are revealed by mapping images of energy dispersive spectrum (EDS) equipped on SEM (Fig. 2B). It can be seen that O, N, and Fe elements well distribute in a large number of Au signals, confirming the formation of Au–PB nanocomposites (Fig. 2C–F).

**Fig. 3:**
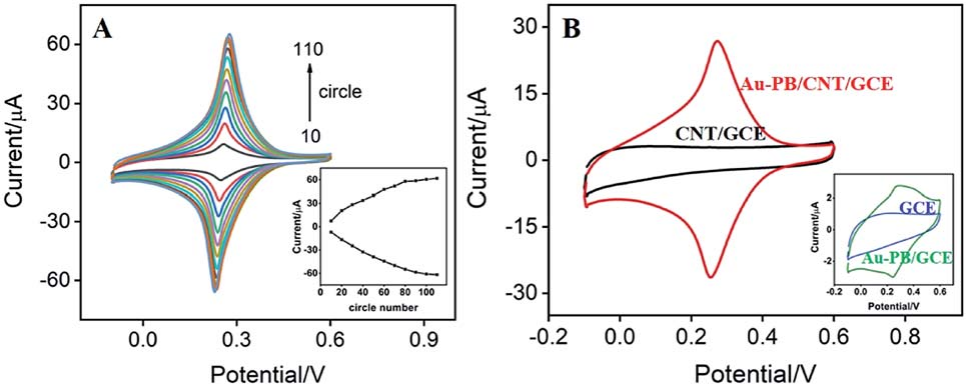
(A) CVs of Au–PB/CNT/GCE prepared with different electro-deposition circles (the increase step of scan cycle is 10 cycles). Inset: dependence of peak current on electro-deposition circles. (B) CVs of Au–PB/CNT/GCE and CNT/GCE. Insets are CVs of Au–PB/GCE and GCE. The supporting electrolyte is KNO_3_ (0.1 M pH 7.0). Scan rate is 100 mV s^−1^.

Scan cycle of electrodeposition is an important experimental condition for the electrodeposition of PB. The effect of this parameter on the electrochemical signal of the modified electrode was investigated. As shown in Fig. 3A, the as-prepared electrode presents a redox pair that is the typical feature resulting from conversion of PB (K[Fe^3+^Fe^2+^(CN)_6_]) and Prussian white (PW, K_2_[Fe^2+^Fe^2+^(CN)_6_]). In addition, the redox current of PB gradually increases with increasing the number of scan cycle. When it turns to more than 90 s, the electrochemical signal no longer changes significantly. Considering the deposition time and the growth rate of electrochemical signal, 30 scan cycles were chosen for the electrochemical deposition of PB (inset in Fig. 3A). As revealed in Fig. 3B, the as-prepared Au–PB/CNT/GCE exhibits typical redox peaks of PB in comparison with the control CNT/GCE. It is worth noting that Au–PB/CNT/GCE presents a significantly higher current than PB/GCE, suggesting the effective signal amplification of CNT (inset in Fig. 3B). The phenomenon might be ascribed to the increased amount of PB in CNT modified GCE.

### 3.2. Improved stability of PB by pDA layer

**Fig. 4:**
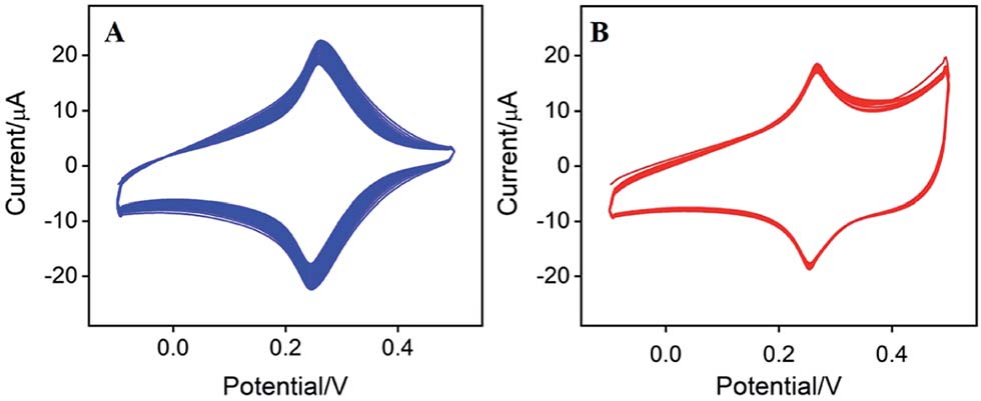
CVs of (A) Au–PB/CNT/GCE or pDA/Au–PB/CNT/GCE (B) in KNO_3_ (0.1 M, pH 7.0) for 20 continuous scans. Scan rate is 50 mV s^−1^.

The biggest challenge for label-free electrochemical biosensors based on surface-confined redox mediator is the instability of electrochemical signal. Low stability from leakage of the PB from the electrode surface is serious limitation for its practical application. In order to evaluate the stability of Au–PB modified electrode, continuous CV scanning was performed on the electrode. As shown in Fig. 4A, the peak current of Au–PB/CNT/GCE significantly reduces by 23.2% when twenty consecutive scan cycles are performed. This fully indicates that the electrodeposited PB is easy to fall off, suggesting poor stability. To overcome this problem, we modified the surface of Au–PB/CNT/GCE by polydopamine layer through *in situ* polymerization of dopamine. It is well known that pDA is the main component of mussels. This biocompatible material can be conveniently prepared by oxidative self-polymerization of dopamine in alkaline solution.^21^ The effect of the self-polymerization time of dopamine on the stability of the as-prepared electrode was investigated (Fig. S2 in ESI†). When the electrode obtained at different dopamine polymerization time was continuously scanned, the increase of the polymerization time firstly decreases the reduction of PB's peak current. When the dopamine polymerization time reaches 30 min, the reduction of PB's peak current no longer changes, indicating improved stability of surface-confined PB by coating of pDA. Thus, the self-polymerization time of dopamine was set at 30 min. The resulting pDA/Au–PB/CNT/GCE exhibits only a slight signal decrease (7.5%) after 20 consecutive scans, indicating good stability (Fig. 4B). Thus, the stability of PB can be significantly improved by coating Au–PB nanocomposite with pDA layer. This strategy has great potential for the construction of label-free electrochemical biosensors.

**Fig. 5:**
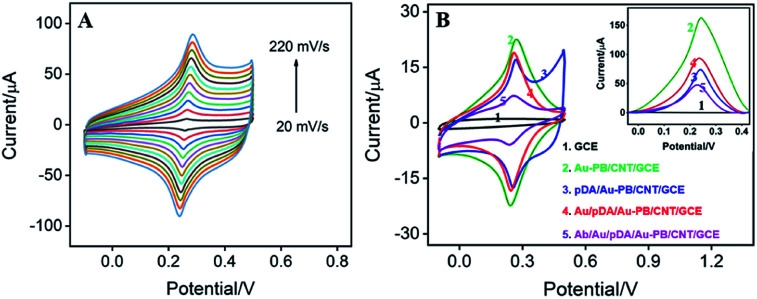
(A) CVs of pDA/Au–PB/CNT/GCE at different scan rates (the increase step of scan rate is 20 mV s^−1^). (B) CV or DPV (inset) of different electrodes in KNO_3_. The scan rate for CV measurement is 50 mV s^−1^.

Fig. 5A illustrates the CVs of pDA/Au–PB/CNT/GCE at different scan rate (*ν*). As seen, the anodic and cathodic peak currents are symmetric. Additionally, both anodic (*I*_pa_) and cathodic (*I*_pc_) peak currents are proportional to the scan rate (*I*_pa_ = 0.2142*ν* + 0.5514, *R*^2^ = 0.9942, *I*_pc_ = −0.3042*ν* + 2.629, *R*^2^ = 0.9983). Thus, pDA/Au–PB/CNT/GCE possesses a reversible and surface-controlled electrochemical process. It is worth noting that with increasing the scan number, the peak potentials of the anode and cathode do not change, suggesting a high electron transfer rate. This is mainly attributed to the promoted electron transfer by the supporting CNT and co-deposited AuNPs.

### 3.3. Fabrication of the immunosensor

In addition to stabilizing the inner layer of PB, pDA layer can also be used as an excellent reductive agent for *in situ* reduction of HAuCl_4_ to further deposit AuNPs,^22–25^ which can be employed for the attachment of antibodies. This process is also proven by the electrochemical signal of PB on the surface of electrode. As illustrated in Fig. 5B, the current signal of PB decreased after coating pDA, while this signal increased again after the deposition of Au NPs on the pDA modified electrode. This is due to the improved electron transfer performance by the introduced AuNPs. Then, recognitive antibodies can be further immobilized onto the outmost AuNPs surface based on the interaction between Au NPs and the –SH or –NH_2_ groups of some amino acid residues in the antibodies. Owing to poor electron transport efficiency of proteins, both the immobilization of antibodies and subsequent BSA blocking of nonspecific sites all lead to a decreased PB signal (Fig. 5B).

Electrochemical impedance spectroscopy (EIS) is an effective tool for monitoring the characteristics of electrode interfaces. Therefore, we also use EIS to characterize the stepwise assembly process for the fabrication of immunosensors. EIS curves of different electrodes are displayed in Fig. S1 (ESI).† As shown, except for the electrode that binds to anti-CEA antibody, the semi-circular diameters of the other electrodes are very small, indicating low interfacial electron transfer resistance (*R*_et_). As revealed in the enlarged view of the EIS curves in the low-frequency region (inset in Fig. S1 in ESI†), the very small *R*_et_ of GCE reduces after the modification of CNT and Au–PB owing to high conductivity of CNT and AuNPs. Though the semi-circular diameter slightly increases after introduction of pDA, it decreases with the formation of the second layer of AuNPs. The significant increase of *R*_et_ after the binding of anti-CEA antibody followed with BSA blocking might be attributed to the nonconductive property of protein, which would obstruct the electron transfer of the redox probe. Thus, successful fabrication of the immunosensor with the recognitive interface is realized.

**Fig. 6:**
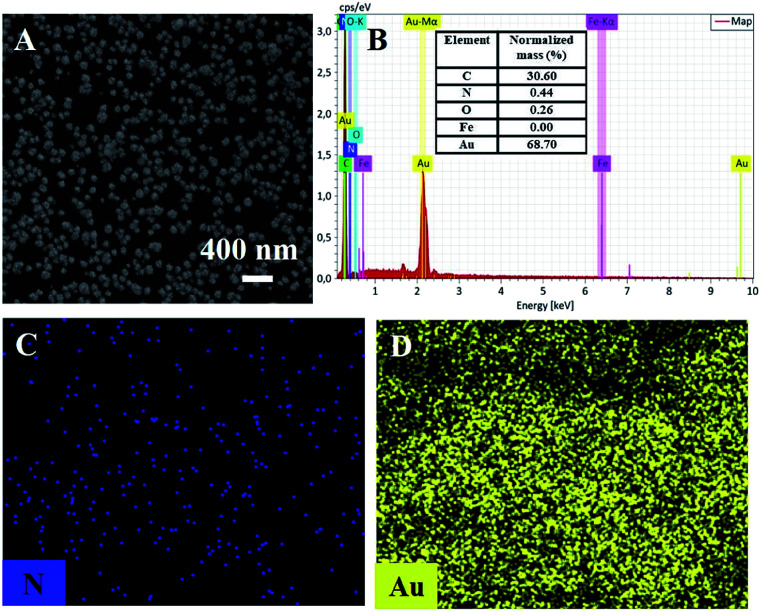
SEM images (A), EDS (B) and (C and D) elemental mapping images of Au/pDA/Au–PB/CNT/GCE.

The morphology of the electrochemically deposited second layer of Au nanoparticles was revealed by SEM. As shown in the Fig. 6A, Au nanoparticles are uniformly distributed on the pDA layer. Elemental analysis and mapping showed the presence of C, N, O, and Au atoms (Fig. 6B–D). Amongst, C and N atoms are from polydopamine. It is worth noting that iron signal is not detected on Au/pDA/Au–PB/CNT/GCE, indicating the coating and protecting effects of pDA layer on Au–PB. Thus, the second layer of Au nanoparticles is necessary for the immobilization of the recognitive antibody.

### 3.4. Reagentless detection of CEA by the immunosensor

**Fig. 7:**
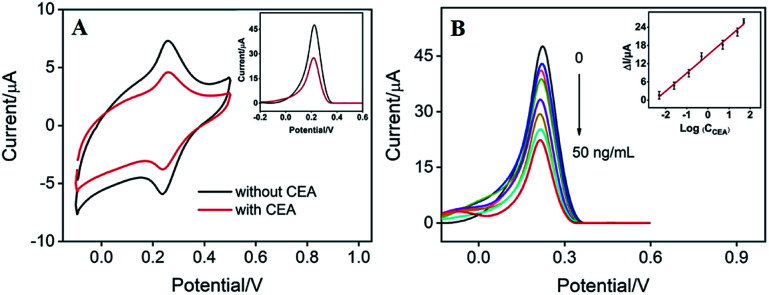
(A) CVs or DPV (inset) of Ab/Au/pDA/Au–PB/CNT/GCE in absence or presence of CEA (25 ng mL^−1^). (B) DPV curves of Ab/Au/pDA/Au–PB/CNT/GCE towards different concentrations of CEA. (B) The linear relationship between the DPV peak current and the logarithm of concentration of CEA.

The detection mechanism of label-free electrochemical immunosensor lies in the reduction of signal of mediator caused by the binding of antigen on recognitive interface. As the surface-confined PB on the electrode can serve as signal indicator, it is not necessary to add extra solution with electrochemical probe. Therefore, reagentless detection of CEA can be realized by the sensor. As shown in Fig. 7A, the binding between CEA and the recognitive antibody on the immunosensing interface causes significant decrease in PB signals measured by CV and differential pulse voltammetry (DPV) due to the increased insulation on the electrode surface by the formation of CEA–antibody composite. The DPV peak current of the as-prepared immunosensor is related to the log value of CEA concentration. As shown in the inset of Fig. 7B, the sensor is able to determine CEA ranged from 0.005 ng mL^−1^ to 50 ng mL^−1^ (*I* = 5.978 log *C* + 14.87, *R*^2^ = 0.9900) with a detection limit (DL) of 3.3 pg mL^−1^ (at a signal-to-noise ratio of 3). The DL value is much lower than the warning CEA level of normal people (usually 5 ng mL^−1^) in serum.

Comparison between detection of CEA using different immunosensor is provided in Table S1.† The LOD is lower than those obtained using Ab/Ag_2_S@ZnO/AuNPs,^26^ Ab/chitosan-AuNPs/GCE,^27^ Ab/chitosan glutaraldehyde,^28^ TiO_2_-AuNPs-carbon paste electrode,^29^ or Ab/AuNPs/Au electrode,^30^ but higher than that obtained from Ab/Ag–Co_3_O_4_@N-doped graphene oxide/GCE,^31^ or Ab/polyethyleneimine/AuNPs@Nafion/K_3_Fe(CN)_6_@chitosan/GCE.^32^ In comparison with other technologies, our electrochemical sensing is attractive because of high sensitivity, no need of bulky instrumentation and easy operation.

### 3.5. Selectivity, stability, reproducibility of the immunosensor and real sample analysis

The selectivity of the immunosensor was studied in CEA (10 ng mL^−1^) solution containing some possible interferences. The biological molecules including glucose (2 mmol L^−1^), bovine serum albumin (BSA, 0.2 mg mL^−1^) or other tumor biomarker (prostate specific antigen, PSA, 50 ng mL^−1^) were investigated. The effect of these possible interference was evaluated using the current ratio (*I*_1_/*I*), which was calculated by comparing the perk current obtained in the presence (*I*_1_) or absence (*I*) of one of the interferences. Though the concentration of the tested interference substances is significantly higher than that of CEA, the current ratio ranges from 0.98 to 1.03, indicating good selectivity of the developed immunosensor.

The response of this label-free immunosensor towards CEA (10 ng mL^−1^) retained 93.2% after 30 days of storage in a refrigerator (4 °C). To evaluate electrode-to-electrode reproducibility, five electrodes were prepared under the same conditions independently. The response to 10 ng mL^−1^ of CEA exhibits an RSD of 2.9%. The good stability and high reproducibility of the immunosensor could be ascribed to the protection effect of pDA and the non-covalent immobilization of antibody.

The application of the as-prepared immunosensor for reagentless detection of CEA in serum was investigated. As demonstrated in Table 1, standard addition method is employed to determine the artificial CEA concentrations by spiking a defined amount of CEA into the serum samples. The recoveries ranges from 95.2–109.8% and the relative standard deviations (RSD) are no more than 3.2%. This reagentless detection of CEA using the developed label-free immunosensor is simple, easy-operation, and of low cost.

**Table 1:** Detection of CEA in serum samples

Sample^a^	Concentration of CEA (ng mL^−1^)		RSD (*n* = 3, %)	Recovery^b^ (%)
	Added	Determined		
1	0.125	0.119	3.2	95.2
2	5.00	5.49	2.6	109.8
3	25.00	26.90	2.5	107.6

a Serum is diluted by a factor of 50.

b Recovery = determined concentration/added value × 100%.

## 4. Conclusions

A label-free electrochemical immunosensor is developed for reagentless detection of tumour biomarker. The multilayer structures on carbon nanotubes modified glassy carbon electrode include co-deposited Au NPs-Prussian blue nanocomposite layer, *in situ* polymerized polydopamine layer, and the second layer of Au NPs for non-covalent binding of recognitive antibody. The developed immunosensor is advantageous in terms of facile preparation, good stability and high sensitivity owing to (1) promoted electrical conductivity of PB by the supporting carbon nanotubes and co-deposited Au NPs; (2) improved stability of PB by the coating of polydopamine; (3) high recognitive ability of antibody owing to non-covalent immobilization strategy. The developed strategy has great potential in the fabrication of label-free electrochemical immunosensor for bioanalysis or medical assay.

## Conflicts of interest

There are no conflicts to declare.

## Supplementary Material

RA-010-D0RA06751B-s001
