# Supplementary material for: Reagentless and sensitive determination of carcinoembryonic antigen based on a stable Prussian blue modified electrode
Source: RSC Adv. 2020 Oct 16;10(63):38316–22. doi: 10.1039/d0ra06751b (PMC9057263; doi:10.1039/d0ra06751b)
Supplement: RA-010-D0RA06751B-s001 [file RA-010-D0RA06751B-s001.pdf]

Electronic Supplementary Material (ESI) for RSC Advances.
This journal is © The Royal Society of Chemistry 2020

Supplementary materials

Figures

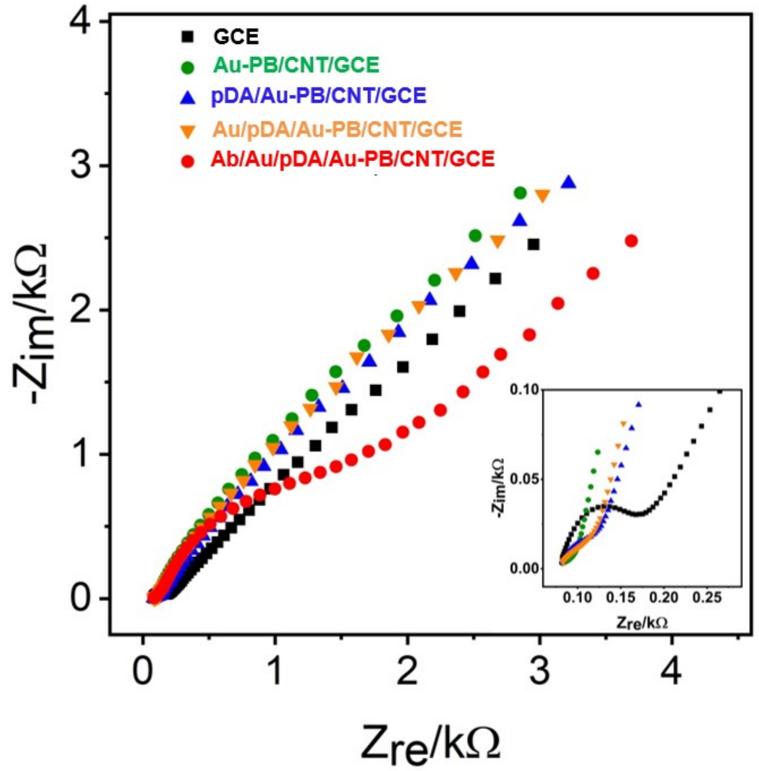

Fig. S1 EIS of different electrodes measured in $Fe(CN)_6^{3-/4-}$ (2.0 mM) containing 0.1 M KCl. The inset shows the enlarged view of the EIS curves obtained on different electrodes (except for Ab/Au/pDA/Au-PB/CNT/GCE) in the low-frequency region.

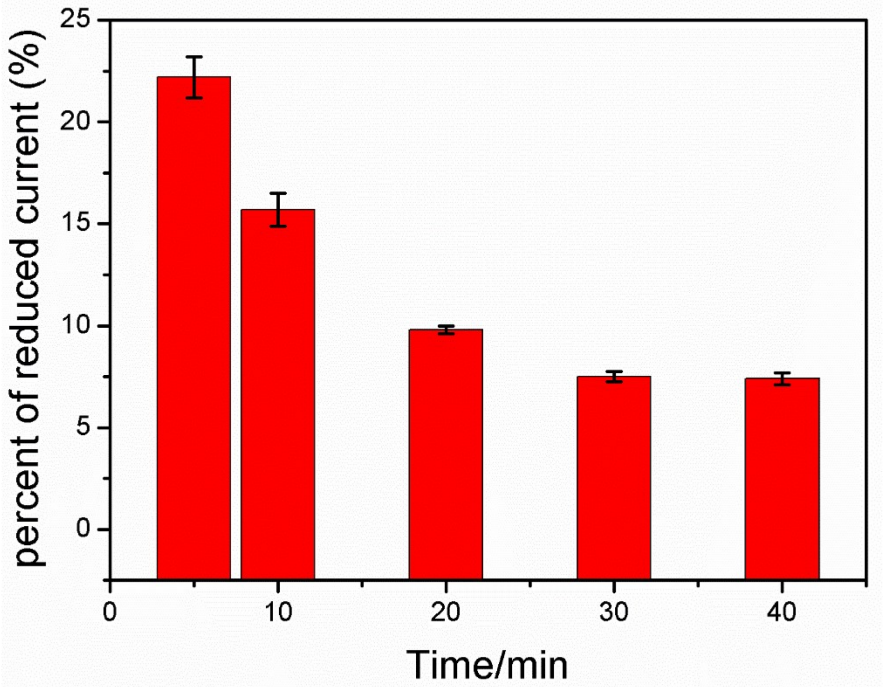

Fig. S2 The percent of reduced current (%) obtained by 20 continuous scans on pDA/Au-PB/CNT/GCE prepared using different polymerization time of dopamine.

Tables

Table S1. Comparison of different sensors for CEA detection

**Sensing element**	**Method**	**Linear range (ng/mL)**	**LOD (pg/mL)**	**Ref.**
Ab/Ag_2S@ZnO/AuNPs	colorimetry	0.1-20	50	[26]
Ab/chitosan-AuNPs/GCE	electrochemistry	0.5-60	100	[27]
Ab/chitosan glutaraldehyde	colorimetry	0.1-20	30	[28]
Ab/Ag-Co_3O_4@N-doped graphene oxide/GCE	electrochemistry	0.001-200	0.18	[31]
TiO_2-AuNPs-carbon paste electrode	electrochemistry	0.01-20	10	[29]
Ab/polyethyleneimine/AuNPs@ nafion/$K_3Fe(CN)_6$@chitosan/GCE	electrochemistry	0.01-150	3	[32]
Ab/AuNPs/Au electrode	electrochemistry	0.5-20	100	[30]
Ab/Au/pDA/Au-PB/CNT/GCE	electrochemistry	0.005-50	3.3	this work
